# Advances on circRNAs Contribute to Carcinogenesis and Progression in Papillary Thyroid Carcinoma

**DOI:** 10.3389/fendo.2020.555243

**Published:** 2021-01-21

**Authors:** Xiaoqin Xu, Jiexian Jing

**Affiliations:** Department of Etiology, Shanxi Cancer Hospital, Taiyuan, China

**Keywords:** papillary thyroid carcinoma, circRNA, characteristics, ceRNA, signaling pathway, diagnosis, prognosis

## Abstract

In view of the highly increased prevalence of papillary thyroid carcinoma (PTC) year by year, it is of great importance to explore new molecular targets for anticancer strategies. Emerging evidence indicates that circular RNAs (circRNAs), characterized by a closed-loop structure and high stability, play important roles in tumorigenesis and development of human cancer by regulating multiple complex biological processes, such as cellular proliferation, metastasis, and metabolism. A comprehensive understanding of the roles of circRNAs will facilitate the development of promising future therapeutic strategies for treating cancers, including PTC. In this paper, we review the profile of circRNA in PTC, its regulatory roles, and the pathological mechanism as well as their related clinical significance. In addition, challenges of this specific field are discussed.

## Introduction

Thyroid cancer (TC) is the most prevalent endocrine malignancy, accounting for nearly one third of the total head and neck malignancies globally (1, 2). Among all cases, 80%–85% of them are papillary thyroid carcinoma (PTC) (3). Although the overall 5-year survival rate of PTC can reach 97%, the 5-year survival rate of patients with advanced PTC is only 59% (4). PTC can still be life threatening and causes poor prognosis due to its invasiveness and metastasis. Extensive efforts have been conducted on research of the carcinogenesis, progression, and effective therapeutic methods of TC. Despite advances in clinical management, including surgery, radiotherapy, levothyroxine treatment, and target therapy, promising and optimal molecular therapies remain to be further explored. In addition to the DNA mutations, such as the BRAFV600E mutation, which was discovered previously, accumulating evidence indicates that non-coding RNAs (ncRNAs) also participate in the progression and pathogenesis of PTC (5–7). Among them, circular RNAs (circRNAs) have attracted increasing attention. Optimistic exploration of PTC-related circRNA likely will be beneficial to pave the way to improve clinical management.

CircRNAs are a newly identified subclass of ncRNA family, and they are produced cotranscriptionally by the spliceosome at the expense of canonical mRNA isoforms, forming a head-to-tail backsplice characterized by a covalently nonlinear, closed-loop structure that lacks either 5’ to 3’ polarity or a polyadenylated tail (8). Based on the biogenesis of circRNAs in human cells, they are usually classified into three types: exonic circRNAs (ecircRNAs), which are generated from the exons of pre-mRNAs; intronic circRNAs (ciRNAs), which are produced from the intronic region in the pre-mRNAs; and exon-intron circRNAs (EIciRNAs), which consist of both exons and introns from the pre-mRNAs. Due to their closed structures, circRNAs are resistant to RNA degradation and more stable than linear RNA. Emerging evidence shows that dysregulation of circRNAs play important roles in promoting tumorigenesis and tumor progression (9). It is demonstrated that circRNAs serve as competitive endogenous RNAs (ceRNAs) or microRNA sponges, compete with microRNAs (miRNAs), and consequently regulate the target gene expression (10). Furthermore, circRNAs are also involved in various physiological and pathophysiological processes, such as modulating alternative splicing (11) and regulating protein–RNA interactions (12). Previous research have profiled the circRNAs expression of PTC and have found significantly differentiated circRNAs in PTC compared with normal thyroid tissue, which may be involved in the pathogenesis of PTC. In the following sections, we highlight the results of recent research efforts, including the profile of markedly dysregulated circRNAs and their related regulatory networks and clinical significance in PTC as well as the current challenges in the field.

## Profiled circRNAs and Its Role in PTC

### Expression and Biological Function of circRNAs in PTC

To date, many different circRNAs have been found either upregulated or downregulated in PTC tissues compared with matched adjacent normal tissues (**Table 1**). In line with tissue expression level, most PTC-related circRNAs are dysregulated in corresponding PTC cell lines versus in normal thyroid cell lines. Based on gain- and loss-function experiments *in vivo* and *in vitro*, each identified circRNA displays significantly altered tumor cell biological behavior or cell phenotype in PTC cell lines, such as cell proliferation, cell cycle, apoptosis, migration, and invasion (**Table 1**), suggesting that the particular circRNA may act as an oncogenic driver or a tumor suppressor. Take cell-cycle regulation as an example; knockdown of circRASSF2, circFNDC3B, and circFOXM1 caused, respectively, significant G1 phase cell-cycle arrest of TPC-1 cells (*p* < 0.01, *p* < 0.01, and *p* < 0.01, respectively). Silenced circRNA_102171 caused G2 phase arrest, and si-circ_0004458 displayed S phase reduction. In contrast, enhanced circRASSF2 expression increased the G2 phase percentage and decreased the G1 phase percentage of K1 cells (*p* < 0.01). Overexpression of circFNDC3B increased the S-phase percentage and decreased the G0/G1 phase percentage of K1 cells (*p* < 0.01). circFOXM1 expression increased the S phase percentage and decreased the G0/G1 phase percentage of K1 cells (*p* < 0.01). On the basis of this series of functional experiments, circRNAs were confirmed to play oncogenic or inhibitory roles in PTC.

**Table 1:** Dysregulated circRNAs and their biological function in PTC.

circRNA	circRNAID	chromosomelocation		Length	Host gene	Function	Tissues (T/N)	reference
Upregulation								
circRASSF2	hsa_circ_0059354	chr20:4760668-4766974		4418nt	RASSF2	cell proliferation and cell apoptosis	112pairs	(13)
circFNDC3B	hsa_circ_0006156	chr3:171965322-171969331		526nt	FNDC3B	cell proliferation and cell apoptosis	42pairs	(14)
circFOXM1	hsa_circ_0025033	chr12: 2966846-2983691		3410 nt	FOXM1	cell proliferation, clone-forming, apoptosis, migration and invasion	78pairs /20 pairs	(15, 16)
hsa_circ_0058124	hsa_circ_0058124	chr2:216270960-216274462		864nt	FN1	cell proliferation, tumorigenicity, tumor invasion, and metastasis	92pairs	(17)
hsa_circ_0039411	hsa_circ_0039411	chr16:55523562-55540586		4418nt	MMP2	cell growth, migration, invasion and cell apoptosis	46pairs	(18)
circBACH2	hsa_circ_0001627	chr6: 90959407–90981660		2995nt	BACH2	cell proliferation, migration and invasion	40pairs	(19)
circRAPGEF5	hsa_circ_0001681	chr7: 22330793–22357656		516nt	RAPGEF5	cell proliferation, migration, and invasion	30pairs	(20)
Has_circ_0008274	hsa_circ_0008274	chr11: 96485180-96489456		244nt	UGGT2	cell proliferation and invasion	142pairs	(21)
circEIF6	hsa_circ_0060060	chr20:33867368-33872594		799nt	EIF6	autophagy, cell apoptosis	6pairs	(22)
circZFR	hsa_circ_0072088	chr5:32379220-32388780		693nt	ZFR	cell proliferation, migration and invasion	41pairs	(23)
circRNA_102171	–	–		309nt	SMURF2	cell proliferation, migration and invasion, apoptosis	47pairs	(24)
circNUP214	hsa_circ_0089153	chr9:134011326-134022971		1102nt	NUP214	cell proliferation, invasion, migration and tumorigenesis	30pairs	(25)
hsa_circ_0004458	hsa_circ_0004458	chr8: 18656804-18662408		448nt	PSD3	cell proliferation, cycle, and apoptosis	48pairs	(26)
circ-0103552	hsa_circ_0103552	chr15:43294752-43314999		920nt	UBR1	cell invasion and migration	56pairs	(27)
circ_0067934	hsa_circ_0067934	chr3:170013698-170015181		170nt	PRKCI	cell proliferation, migration, and invasion and apoptosis	57pairs	(28)
circMAN1A2	–	–		–	–	-	57T/121N	(29)
circNEK6	hsa_circ_0088483	chr9:127055127-127101944		911nt	NEK6	cell growth and invasion	GSE3678 GSE93522	(30)
hsa_circRNA_007148	–	–		–	–	–		(31)
Down-regulation								
circ-ITCH	–	–		–	–	cell proliferation, invasion and apoptosis	14pairs	(32)
hsa_circ_0137287	hsa_circ_0137287	chr8:92301363-92307931		284nt	SLC26A7	–	120T/60N	(33)
hsa_circRNA_100395	–	–		–	–	–		(34)
hsa_circRNA_047771	–	–		–	–	–		(35)

T, Tumor tissue; N, Normal tissue.

### Biogenesis, Stability, and Subcellular Location of Profiled circRNAs in PTC

Many reports demonstrate that circRNAs are spliced and derived from the host genes (**Table 1**), and even some circRNAs may impact the mRNA expression level of their host genes. As shown in **Table 1**, characteristics of circRNAs are represented, including circRNA ID (http://www.circbase.org), chromosome position, spliced length, and host gene. Among them, most circRNAs are classified as ecircRNAs, such as hsa_circ_0006156 (14), Hsa_circ_0058124 (16), CircBACH2 (19), hsa_circ_0001681 (20), CircRNA_102171 (24), and hsa_circ_0004458 (26). Exceptionally, CircNEK6 is a kind of exonic circRNA encoding the mRNA NEK6. In addition, some circRNAs are not found in circBase because of limited information in current reports, including hsa_circRNA_100395, hsa_circRNA_047771, hsa_circRNA_007148, and circ-ITCH, circMAN1A2.

Generally, stability of the circRNA is critical for exerting its function. Analysis of stability for circRNA and its host gene in PTC cells, treated with transcription inhibitor actinomycin D, reveals that the half-life of circRASSF2 exceeds 24 h, whereas that of RASSF2 mRNA is only about 3 h in TPC-1 cells (13). Similarly, the half-life of circFNDC3B and circFOXM1 transcript exceeds 24 h, much more stable than the corresponding host genes FNDC3B and FOXM1 (14, 15), respectively. Furthermore, circRNA is resistant to RNase R digestion. This proves that circRNAs are extremely more stable than their mRNA level. Given their stability, circRNAs are appropriate for future clinical applications for PTC.

In addition, subcellular location may be related to the distinct molecular roles of various kinds of circRNAs in cells. EcircRNAs are predominantly localized in the cytoplasm (35), and ciRNAs and ElciRNAs are preferred in the nucleus (36). Subcellular location by cell fraction assay and FISH analysis indicates that circFNDC3B (14), circBACH2 (19), circRAPGEF5 (20), and circNUP214 (25) are predominantly localized in the cytoplasm of PTC cells, and hsa_circ_0058124 primarily appears in the nucleus and also exists in cytoplasm (17). In brief, it is essential to get the properties of circRNAs to facilitate the following pathological mechanism.

## Pathological Mechanism of circRNAs in PTC

CircRNAs are widely involved in human physiological and pathological processes and can be used in various manners (37), including (1) serving as microRNA (miRNA) or protein sponges; (2) interacting with proteins, such as recruiting specific proteins, enhancing protein function, and functioning as protein scaffolding; and (3) translating into peptides. Highly abundant circRNAs have been found to contain many competing miRNA binding sites. Therefore, they can be used as RNA “sponges” to cooperatively adsorb miRNAs, thereby regulating the expression of downstream target genes that are inhibited by miRNAs through competing with endogenous RNAs (38). In cancer research, the use of circRNAs as miRNA sponges to regulate downstream target genes is widely reported.

### CircRNA Serves as ceRNA Involved in PTC Progression

**Figure 1:**
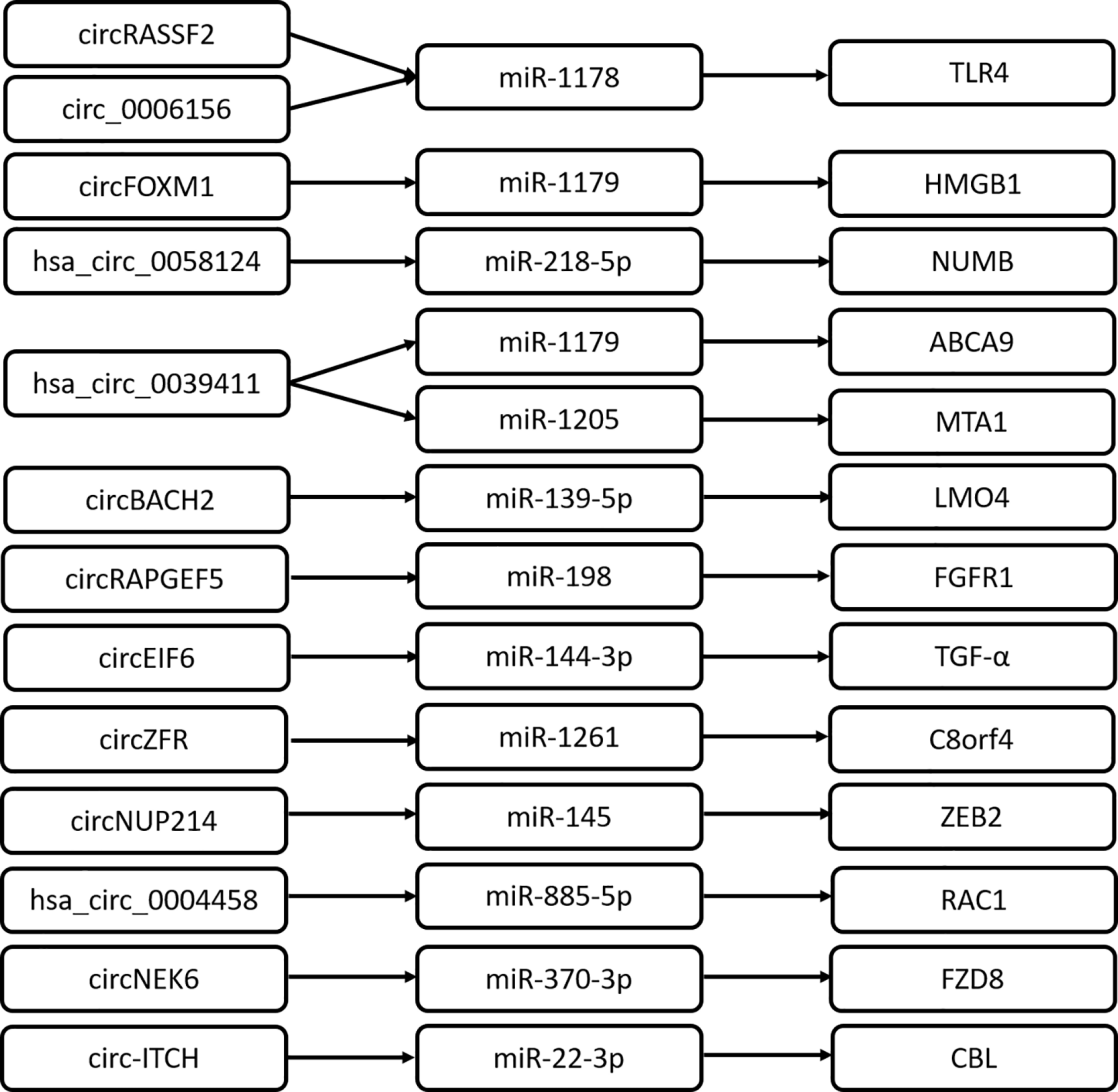
CircRNAs play roles by acting as ceRNA in PTC.

CircRNAs are important transcriptional regulators of gene expression, relieving the association between miRNA and target genes involved in the pathogenesis of various diseases. It is reported that circRNAs could act as miRNA sponges and regulate the expression of downstream target genes. Previous studies show that an increasing number of circRNA/miRNA/mRNA axes are identified to promote PTC progression (**Figure 1**). This well depicts the interactional network between circRNA and RNA for a better understanding of the transcriptional regulation mechanism by circRNA. Of note, some RNA regulatory network mediated by circRNA remains to be further improved. For example, circ_0025033/miR-1231 and miR-1304 (16), circ-0103552/miR-127 (27), hsa_circ100395/miR-141-3p/miR-200a-3p (34).

### Signaling Pathway Modulated by PTC-Related circRNAs

CircRNAs also exert their regulatory roles to modulate signaling pathways in cancer, for instance, the wnt/β-catenin signaling pathway (39–40), AMPK/mTOR signaling pathway (41), PI3K/AKT signaling pathways (42–44), and NOTCH pathway (45, 46). As a classical pathway, the wnt signaling pathway is involved in many phases of vertebrate embryonic development and contributes to tumorigenesis. Its aberrant activation could facilitate the progression of various human cancers. CircRNA_102171 directly interacts with CTNNBIP1 and impairs the formation of CTNNBIP1/β-catenin complex (24). Consequently, circRNA_102171 promotes the interaction of β-catenin with TCF proteins; significantly enhances the expression of corresponding target genes, such as CCND1, CCND2, MYC, and SOX4; and activates the Wnt/β-catenin pathway in a CTNNBIP1-dependent manner (24). Frizzled class receptor 8 (FZD8) is reported to be one of the cell surface receptors of the Wnt signaling pathway, which belongs to the Frizzled family of serpentine proteins. Chen F et al. find that circNECK6 binds target miR-370-3p to inhibit FZD8 degradation and the upregulated FZD8 activates the wnt signaling pathway (30). Wang M et al. reveals a novel mechanism regulating the wnt pathway by circRNA (32). Circ-ITCH sponges miR-22-3p to elevate CBL (an E3 ligase of nuclear β-catenin) expression, which leads to the inactivation of the Wnt/β-catenin pathway and consequently attenuates PTC progression. Moreover, Yao Y et al. reports that hsa_circ_0058124 plays an oncogenic driver in PTC by downregulating the NOTCH3 signaling pathway. hsa_circ_0058124 may exert its biological effects in PTC through hsa_circ_0058124/miR-218-5p/NUMB, subsequently with repression of the NOTCH3/GATAD2A axis because NUMB is a strong suppressor of the NOTCH pathway (17). Collectively, circRNAs modulate various pathways to activate the PTC progression program.

## circRNAs Act as Tumor Biomarkers in PTC

### The Relationship Between circRNAs and Clinicopathological Parameters in PTC

Clinical analysis reveals that dysregulated circRNAs correlate with aggressive clinicopathological characteristics of PTC, including tumor size, TNM stage, lymph node metastasis, T stage, distal metastasis, and extrathyroidal extension (**Table 2**). Among them, highly expressed circRASSF2 (13), circFNDC3B (14), circFOXM1 (15), hsa_circ_0058124 (17), circBACH2 (19), circ_0008274 (21), circZFR (23), hsa_circ_0004458 (26), circ_0067934 (28), and hsa_circRNA_007148 (31) positively correlate with a few aggressive features, whereas lower levels of hsa_circ_0137287 (22), hsa_circRNA_047771 (31), and circ-ITCH (32) negatively correlate with some clinical features. Of note, tumor size is classified by different groups in different research. For example, downregulation of hsa_circ_0137287 correlates with tumor size >2 cm. Upregulated circ_0067934 and circ_0006156 correlate with tumor size >1 cm, hsa_circ_0004458 with tumor size ≥3 cm, hsa_circ_0058124 with tumor size >2 cm, and circFOXM1 with tumor size >3 cm. Generally, PTC is often combined with other types of thyroid disease, such as Hashimoto’s thyroiditis (HT), nodular goiter (NG), and so on. It is reported that the level of circFOXM1 is significantly associated with NG (*P* = 0.009) (15). In addition, a great deal of previous research indicates that the BRAFV600E mutation is identified as an essential genetic factor in PTC progression. The BRAFV600E mutation, which can cause activation of MAPK pathway signaling, is significantly associated with more aggressive characteristics of PTCs and facilitates risk stratification and the management of patients with thyroid nodules. A decreased hsa_circRNA_047771 expression level is associated with the BRAF^V600E^ mutation (*P* < 0.05) (31). Collectively, the association between circRNAs and aggressive clinicopathological characteristics supports that circRNAs can serve as prognostic factors for PTC patients.

**Table 2:** The relationship between circRNAs and clinical features of PTC.

Clinical features	Upregulated circRNAs	Downregulated circRNAs
Tumor size	circFNDC3B (14), circFOXM1 (15), hsa_circ_0058124 (17), hsa_circ_0004458 (26), circ_0067934 (28)	hsa_circ_0137287 (22)
Lymph node metastasis	circRASSF2 (13), circFNDC3B (14), circFOXM1 (15), hsa_circ_0058124 (17), circBACH2 (19), circ_0008274 (21), circZFR (23), hsa_circ_0004458 (26), circ_0067934 (28), hsa_circRNA_007148 (31)	hsa_circ_0137287 (22), hsa_circRNA_047771 (31), circ-ITCH (32)
TNM stage	circFNDC3B (14), circFOXM1 (15), hsa_circ_0058124 (17), circBACH2 (19), circ_0008274 (21), circZFR (23), hsa_circ_0004458 (26), circ_0067934 (28)	hsa_circRNA_047771 (31), circ-ITCH (32)
T stage	circRASSF2 (13), hsa_circ_0004458 (26)	hsa_circ_0137287 (22)
Distal metastasis	circRASSF2 (13), hsa_circ_0004458 (26)	
Extrathyroidal extension	hsa_circ_0058124 (17)	hsa_circ_0137287 (22)

### Diagnostic Value of PTC-Related circRNAs

Pathological diagnosis is a gold standard method for the preoperative evaluation of thyroid nodules; however, cytology remains indeterminate for up to 30% of nodules that cannot be definitively diagnosed (47). Except for the BRAFV600E mutation, a novel molecular biomarker is required in favor of clinical diagnosis and risk stratification, especially for efficient management of cN0 papillary thyroid microcarcinoma (PTMC). Extensive exploration in recent years reveals that ncRNAs, such as miRNAs, lncRNAs, and circRNAs, could function as a promising diagnostic biomarker for PTC patients (48, 49). A receiver operating characteristic (ROC) curve was used to evaluate the diagnostic value of circRNAs in PTC tissues compared with paratumor tissues, and it was found that the area under the ROC curve (AUC) of circFNDC3B was 0.891 (95% CI = 0.820–0.961, *P* < 0.0001) (14) and of circBACH2 was 0.8631 (95% CI = 0.7774–0.9489, *P* < 0.0001) (19). More importantly, circRNAs also serve as postsurgical diagnostic biomarkers. Lan X et al. find that hsa_circ_0137287 has a potential diagnostic value in predicting malignancy (AUC = 0.8973, 95% CI = 0.8452–0.9494, *P* < 0.0001), extrathyroidal extension (AUC = 0.6885, 95% CI = 0.5908–0.7862, *P* = 0.0009), and lymph node metastasis (AUC = 0.6691, 95% CI = 0.5641–0.7742, *P* = 0.0034), respectively (33). Additionally, hsa_circRNA_047771 (AUC = 0.876, 95% CI = 0.78–0.94, sensitivity = 87.5%, specificity = 80.0%) and hsa_circRNA_007148 (AUC = 0.846, 95% CI = 0.75–0.96, sensitivity = 82.5%, specificity = 77.5%) may be candidate diagnostic biomarkers for PTC (31). In view of, so far, limited exploration, further studies are required to discover more optimal biomarkers for diagnosis of PTC.

### Predicting Roles of circRNAs for Prognosis in PTC

Previous follow-up studies indicate that most PTC patients have a good prognosis: 85% of PTC cases are highly curable for innocent biological behavior. However, it is necessary to carefully observe the recurrence and metastasis, especially for advanced PTC patients. As with other coding genes (BRAFV600E, RAS, etc.) and noncoding genes (miRNA, lncRNA, etc.), circRNAs may be potential predictors for prognosis of PTC. Kaplan–Meier survival curve analysis reveals that PTC patients with low expression of circFNDC3B display obviously longer overall survival (OS) times than those with high expression of circFNDC3B (*P* < 0.05) (14). Similar to circFNDC3B, downregulated circBACH2 had relatively longer OS (*P* < 0.05) (19), a higher expression of circZFR in PTC patients is correlated with worse prognosis (23), and patients with high expression of circ_0067934 show lower survival rates (28). Moreover, Cox proportional hazards regression model analysis also indicates that circ_0067934 is an independent risk factor for prognosis (RR = 4.385, 95% CI = 1.087–17.544, *P* = 0.038) (28), like the circ-ITCH as well (32). More importantly, it is necessary to monitor relapse and progression by reliable biomarkers in long-term follow-up studies. In addition, the relationship between circRNAs, such as circFND3B, circBACH2, and circZFR, and prognosis-predicting roles reveals that it is insufficient to confirm its predicting role for prognosis due to limited survival analysis. Maybe it will be more convincing if performing further analysis by Cox proportional hazards regression models. Even the researcher could observe the relationship between circRNA and recurrence and metastasis in PTC for fine management of PTC, to fully elucidate the prognostic value of circRNAs for PTC.

## Challenges and Prospects

To date, a handful of ncRNAs have been identified, and many have shown oncogenic or tumor-suppressive roles in human cancer, especially lncRNAs and circRNAs. However, it is just like the tip of an iceberg. Despite advances in the relationship between circRNAs and PTC, current research still has a few limitations. For example, the sample size and histological types of TC are limited. Except for circEIF6 (22), most TC-related circRNA research does not include other TCs such as anaplastic thyroid carcinoma (ATC) and medullary thyroid carcinoma (MTC) due to their low incidence. However, it is necessary to explore further by prolonging the observation period and performing multicenter clinical studies.

Furthermore, the molecular mechanism of circRNAs in the PTC pathological process needs to be further clarified to establish RNA regulatory networks. Currently, most studies focus on the “molecular sponge” function or ceRNA role of certain circRNAs. According to ceRNA theory, artificial circRNAs engineered with diverse methods can act as potential and promising therapeutic molecular tools. Nevertheless, circRNAs represent diversity in functions. Therefore, other functions of circRNAs in TC should be explored for a more comprehensive landscape and better understanding of the mechanism in the future, such as alternative splicing, regulation of gene transcription, and crosstalk with RBPs. More importantly, it needs a series of sufficient and logically scientific proofs outside of the molecular mechanism research for a reliable but not farfetched explanation.

Additionally, in view of the clinical applications of circRNAs, further studies should pay more attention to evaluating the diagnostic and prognostic value of circRNAs and the associations with clinical drug resistance. Notably, few reports examine PTC-related circRNAs involved in this field. Liu F et al. demonstrates that circEIF6 associates with chemo-resistance (cisplatin-resistance) by influencing cell autophagy (22). More importantly, circRNAs could be secreted into blood, saliva (50), and even exosomes (51), which play important roles in the tumor microenvironment, suggesting that the circRNA level in body liquid and FNAB samples could facilitate clinical management, such as serum circMAN1A2 (29), serum exosomal circRASSF2 (13), and circ_0006156 (14).

Taken together, it is expected to identify more promising RNA signatures and unveil the underlying mechanism of circRNAs for better understanding of the etiology and pathological progress in TC, which sheds light on the potential applications of circRNAs for translational medicines.

## Author Contributions

XX drafted the manuscript. JJ supervised and revised the manuscript. All authors contributed to the article and approved the submitted version.

## Conflict of Interest

The authors declare that the research was conducted in the absence of any commercial or financial relationships that could be construed as a potential conflict of interest.
